# Liver imaging and pregnancy: what to expect when your patient is expecting

**DOI:** 10.1186/s13244-024-01622-x

**Published:** 2024-02-27

**Authors:** Giorgia Porrello, Roberto Cannella, Jacques Bernuau, Antoine Agman, Giuseppe Brancatelli, Marco Dioguardi Burgio, Valérie Vilgrain

**Affiliations:** 1 Service de Radiologie, AP-HP Nord, Hôpital Beaujon, Paris, Clichy France; 2 Department of Biomedicine, Neuroscience and Advanced Diagnostics (Bi.N.D.), University Hospital “Paolo Giaccone”, Palermo, Italy; 3 AP-HP Nord, Hôpital Beaujon, Service d’Hépatologie, Paris, Clichy France; 4 Service de Gynécologie obstétrique maternité, AP-HP Nord, Hôpital Beaujon, Paris, Clichy France; 5 Université Paris Cité, Inserm, Centre de recherche sur l’inflammation, F-75018 Paris, France

**Keywords:** Pregnancy, Liver disorders, Complications, Focal liver lesions, Liver transplant

## Abstract

Liver diseases in pregnancy can be specific to gestation or only coincidental. In the latter case, the diagnosis can be difficult. Rapid diagnosis of maternal-fetal emergencies and situations requiring specialized interventions are crucial to preserve the maternal liver and guarantee materno-fetal survival. While detailed questioning of the patient and a clinical examination are highly important, imaging is often essential to reach a diagnosis of these liver diseases and lesions. Three groups of liver diseases may be observed during pregnancy: (1) diseases related to pregnancy: intrahepatic cholestasis of pregnancy, pre-eclampsia, eclampsia, hemolysis, elevated liver enzymes and low platelets (HELLP) syndrome, and acute fatty liver of pregnancy; (2) liver diseases that are more frequent during or exacerbated by pregnancy: acute herpes simplex hepatitis, Budd-Chiari syndrome, hemorrhagic hereditary telangiectasia, hepatocellular adenoma, portal vein thrombosis, and cholelithiasis; (3) coincidental conditions, including acute hepatitis, incidental focal liver lesions, metabolic dysfunction–associated steatotic liver disease, cirrhosis, hepatocellular carcinoma, liver abscesses and parasitosis, and liver transplantation. Specific knowledge of the main imaging findings is required to reach an early diagnosis, for adequate follow-up, and to avoid adverse consequences in both the mother and the fetus.

**Critical relevance statement** Pregnancy-related liver diseases are the most important cause of liver dysfunction in pregnant patients and, in pregnancy, even common liver conditions can have an unexpected turn. Fear of radiations should never delay necessary imaging studies in pregnancy.

**Key points**

• Pregnancy-related liver diseases are the most frequent cause of liver dysfunction during gestation.

• Fear of radiation should never delay necessary imaging studies.

• Liver imaging is important to assess liver emergencies and for the diagnosis and follow-up of any other liver diseases.

• Common liver conditions and lesions may take an unexpected turn during pregnancy.

• Pregnancy-specific diseases such as pre-eclampsia and HELLP syndrome must be rapidly identified. However, imaging should never delay delivery when it is considered to be urgent for maternal-fetal survival.

**Graphical Abstract:**

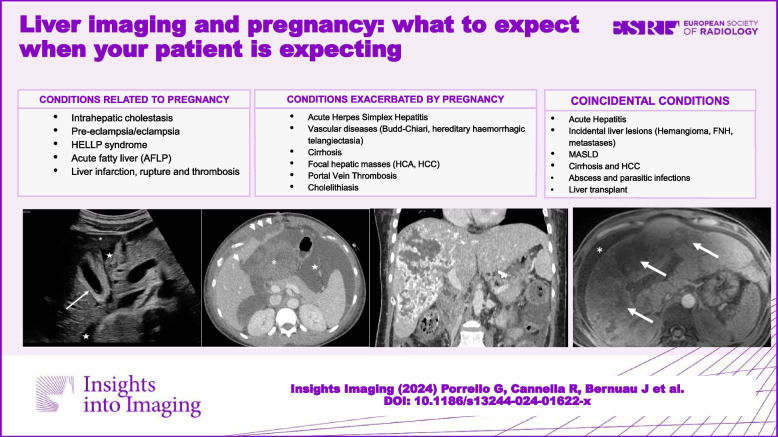

## Introduction

The increased production of estrogens and progesterone in pregnancy affects the metabolic and excretory functions of the liver [1]. Global liver function is primarily modified by hemodynamic and biochemical changes to adapt to the increased metabolic demand. Primary systemic vasodilatation may occur as early as the first trimester, inducing an increase in plasma volume and hyperdynamic circulatory status, with a 30 to 50% increase in cardiac output [2]. All these changes affect liver function. On color Doppler ultrasonography (CDUS), portal blood flow increases while hepatic arterial resistance decreases in the 3rd trimester [3]. There is impaired clearance of substances with extensive hepatic metabolism, and pharmacokinetic changes in the liver [4]. Hemostatic changes lead to a procoagulant state, especially in the second half of pregnancy with increasing clotting factor values and fibrinogen, and a decrease in anticoagulant factors and fibrinolysis [5]. During pregnancy, serum phosphatase alkaline activity increases in the 3rd trimester. Serum aminotransferase, gammaglutyltransferase, and bile acids remain unchanged but serum albumin concentration decreases by hemodilution [6–8].

The overall prevalence of pregnancy-related liver diseases (LD) is higher in low- and middle-income countries, ranging from 3 to 11.3% worldwide [6, 7, 9]. Pregnancy-related LD are the most frequent cause of liver dysfunction during gestation and may have a high risk of mortality for both the mother and fetus, especially in the absence of an early diagnosis and rapid treatment [3, 6–8]. Liver imaging is important to for the diagnosis and follow-up of liver conditions, as well as to determine the specific often urgent treatment.

This review presents an overview of the key imaging findings associated with LD in pregnancy. The following conditions will be discussed: (1) those specifically related to pregnancy, in particular, intrahepatic cholestasis of pregnancy, pre-eclampsia/eclampsia, hemolysis, elevated liver enzymes and low platelets (HELLP) syndrome, and acute fatty liver of pregnancy; (2) conditions that are more frequent during, or exacerbated by, pregnancy: acute herpes simplex hepatitis, Budd-Chiari syndrome, hemorrhagic hereditary telangiectasia, hepatocellular adenoma, portal vein thrombosis, and cholelithiasis; and (3) coincidental conditions including acute hepatitis, incidental focal liver lesions, metabolic dysfunction–associated steatotic liver disease (MASLD), cirrhosis, hepatocellular carcinoma, liver abscesses and parasitosis, and liver transplantation (Table 1). Finally, diagnostic imaging algorithms will be presented to help recognize hepatic complications and guide the diagnostic pathway of pregnant patients.

**Table 1:** Classifications of hepatobiliary disorders according to their relationship with pregnancy

**Related to pregnancy**	**Exacerbated by, or more frequent in, pregnancy**	**Coincidental**
Intrahepatic cholestasis of pregnancy (ICP)	Acute herpes simplex hepatitis	Acute hepatitis
Pre-eclampsia/eclampsia	Budd-Chiari syndrome (BCS)	Hemangioma, focal nodular hyperplasia (FNH), metastasis
Elevated liver enzymes, low platelets (HELLP) syndrome	Hereditary haemorrhagic telangiectasia (HHT)	Metabolic dysfunction–associated steatotic liver disease (MASLD)
Acute fatty liver of pregnancy (AFLP)	Hepatocellular adenoma and HCC (increased risk of tumor growth and rupture)	Cirrhosis and HCC
Liver infarction, rupture, and thrombosis	Portal Vein Thrombosis	Abscesses and parasitic infections
	Cholelithiasis	Liver transplantation

## Liver imaging perspective

Liver imaging during pregnancy helps guiding the differential diagnoses and identifies parenchymal or vascular complications and emergencies. Ultrasonography (US) is usually the first-line test. It is accessible, reliable, and safe even during the first trimester, as long as the use of Doppler is limited [10]. Recent studies have confirmed that contrast-enhanced US (CEUS) is safe and can therefore be used to evaluate the vascularity of single lesions [11]. Standard magnetic resonance imaging (MRI) (e.g., ≤ 3-T MRI) may be safely used in all trimesters [10–13] as an alternative. This technique provides greater spatial and soft tissue resolution, multiplanar images, and larger fields of view [14, 15]. Gadolinium-based contrast administration is still controversial. Although this agent crosses the placenta, no direct toxic effects have been identified. However, the number of studies in humans is limited [16], thus it should only be used when strictly necessary [10, 13, 14, 17], and it should not be used in patients with renal impairment [17]. Macrocyclic contrast agents (e.g., gadobutrol, gadoterate meglumine) are recommended because they are associated with the lowest risk of nephrogenic systemic fibrosis [17]. Hepatobiliary contrast agents have an intermediate risk of nephrogenic systemic fibrosis. In particular, high doses have been shown to be teratogenic and toxic in animal studies; thus, these agents must only be administered when there is no alternative and the potential benefits justify the potential risks [10, 18].

### Radiation exposure

Ionizing radiation exposure is a common concern during pregnancy. However, if standard dose-reducing precautions are taken, the absolute risk of anomalies or abortion is negligible at doses < 50 mGy, even if the fetus is included in the field of view [10, 19]. Doses < 50 mGy may only have an “all‐or‐none” effect before implantation, in the first 2 weeks of gestation [20].

Between 50 and 100 mGy, the theoretical risks are uncertain [9]. There is a risk of teratogenicity between weeks 2 and 8 [20] and a possibility of embryonic demise within the first 2 weeks alone [19]. At doses > 100 mGy, spontaneous abortion, malformations, and neurological anomalies may occur, especially between 8 and 17 weeks of gestation, during organogenesis and the development of the nervous system [13, 19]. One computed tomography (CT) scan of the abdomen and pelvis represents a dose of 5.76 ± 3.22 mGy [16, 21], depending on the machine and dose-reducing protocols. Therefore, in a single study, the fetus will probably be exposed to < 50 mGy, even if multiple phases are acquired [10, 16]. Thus, fear of radiation should never delay imaging, especially in emergencies. The main precaution is to keep doses as low as reasonably achievable [10, 13, 16, 19]. At present, there is very little information in the literature on the use of techniques such as dual energy, spectral CT, or ultralow doses, which would further reduce doses.

Iodine-based intravenous contrast agents are safe [16–19]. Although there have been concerns about possible damage to the fetal thyroid, studies with lower-osmolality/non-ionic contrast media have not shown any teratogenic effects or induction of hypothyroidism [16–19].

A summary list of imaging indications is presented in Table 2.

**Table 2:** Classification of hepatobiliary disorders according to clinical and imaging classifications

Condition	Trimester	Symptoms	Imaging Findings	Main Role of Imaging	Imaging technique
ICP	II-III	Pruritus, increased serum bile acids, jaundice uncommon	Gallstones (without intrahepatic biliary dilatation) and/or intrahepatic lithiasis	Differential diagnosis; detection of gallstones and complications	US +++ MRCP ++
AFLP	II - III	Recent nausea and/or vomiting, polydipsia, jaundice, abdominal pain	Fatty changes, ascites	Diagnosis, assessment of complications	US ++ CT + (+++ if complications) MRI +++
Pre-eclampsia/ Eclampsia	II-III	Arterial hypertension, edema	Portal vein thrombosis, hemorrhage	Assess liver involvement as soon as possible	US ++ CT ++
HELLP Syndrome	III/post-partum	Frequent arterial hypertension, right upper quadrant pain, fever (associated with liver infarct), edema, hypovolemic shock	Infarct, hemorrhage, subcapsular hematoma, liver rupture	Diagnosis and assess complications as soon as possible	US ++ (bedside patient) CT +++ (if unstable)
Budd-Chiari Syndrome	II - III	Abdominal pain, ascites, hepatomegaly, liver failure	Hepatic venous occlusion, regenerative nodules, hepatomegaly, portal hypertension	Detect venous thrombosis, surveillance	US +++ CEUS ++ CT + MRI +
HHT	III/post-partum	Pain, liver failure, bleeding	Liver infarction, aneurysm rupture	Surveillance and diagnosis of complications	US ++ CT + (+++ if complications) MRI +
Cirrhosis	Any	Decompensation, worsening portal hypertension, variceal bleeding	Ascites, focal liver lesions assessment, portal hypertension	Surveillance, assess complications, treatments (TIPS)	US +++ CEUS ++ (if lesions) MRI ++ (if lesions)
Focal Hepatic Masses	Any	Abdominal tension or pain / Asymptomatic	Growth +/- bleeding and liver rupture	Detect and characterize	US ++ CEUS ++ MRI ++ CT + (only if cancer)
Cholelithiasis	Any	Right upper quadrant pain, nausea	Gallstones, pancreatitis, ductal dilatation	Diagnosis	US +++ MRI ++
Hepatitis	Any	Nausea, inconstant jaundice, liver dysfunction	Hepatomegaly, gallbladder wall thickening, diffuse hepatic hypoechogenicity // small hypoechoic lesions	Differential diagnosis and confirmation of diagnosis	US +++ CT + MRI ++
Abscesses	Any	Fever, nausea, pain	Avascular, thick-walled fluid collection, perfusion disorders	Diagnosis and guidance of treatment	US +++ CT + MRI ++
Liver transplantation	2nd half of pregnancy //post-partum	Pre-eclampsia and HELLP syndrome	Infarct, hemorrhage, hematoma, liver rupture	Assess complications as soon as possible	US +++ CT (when complications) +++

*Abbreviations*: *ICP* intrahepatic cholestasis of pregnancy, *AFLP* acute fatty liver of pregnancy, *HELLP* hemolysis, elevated liver enzymes, low platelet, *HHT* hereditary hemorrhagic telangiectasia, *TIPS* trans-jugular intrahepatic portosystemic shunt, *US* ultrasonography, *CEUS* contrast-enhanced US, *CT* computed tomography, *MRI* magnetic resonance imaging, *MRCP* magnetic resonance cholangiopancreatography. Indication for imaging techniques is scored from + to +++

## Pregnancy-related liver disorders

### Intrahepatic cholestasis of pregnancy

**Fig. 1:**
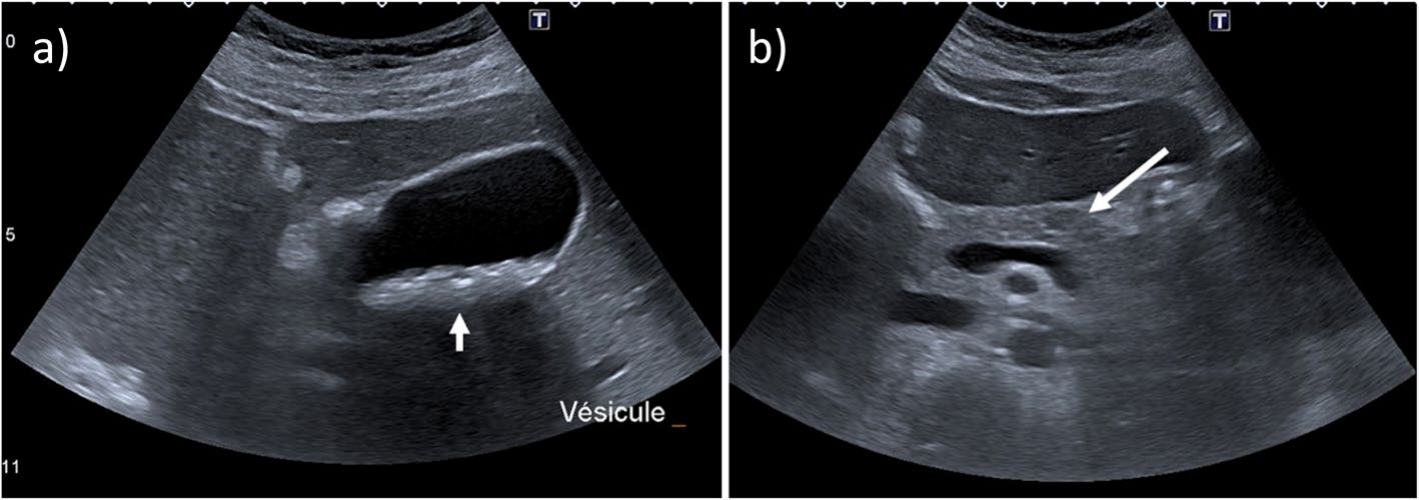
Intrahepatic cholestasis of pregnancy in a 36-year-old woman. **a** Transverse abdominal US scan shows multiple gallstones (short arrow) into the gallbladder. Contextual transverse scan of the pancreas (**b**) reveals the presence of a blurred hypoechoic lesion on the pancreas that (arrow), together with laboratory evidence of raised amylase and lipase, led to the diagnosis of acute pancreatitis

Intrahepatic cholestasis of pregnancy (ICP) is a symptomatic cholestasis that affects 0.1–4% of pregnancies and usually develops during the third trimester [9, 22, 23], although it may occur earlier, such as at the end of the first trimester [9]. ICP is diagnosed by excluding other cholestatic diseases and is characterized by worsening pruritus, increased serum aminotransferase activities, and elevated serum bile acids. When the latter exceeds 40 μmol/L (severe ICP), the risk of preterm labor, fetal asphyxia, or stillbirth increases, with a significant increase above 100 μmol/L [23]. Gallstones are observed in 13–20% of cases, usually associated with a low phospholipid–associated cholelithiasis syndrome [24]. US and MR cholangiopancreatography (MRCP) may be performed to demonstrate intra- and/or extrahepatic cholelithiasis (Fig. 1) [22]. Ursodeoxycholic acid is prescribed because it may delay a premature delivery [22, 23]. Clinical and biochemical resolution occurs within 6 weeks after delivery [7–9, 22, 23]. ICP recurs in genetic forms that may be associated with bile acid transporter mutations [22].

### Pre-eclampsia

Pre-eclampsia (PE) is a progressive systemic disorder that affects 3–10% of all pregnancies [25] and is defined by the association of new-onset arterial hypertension and proteinuria, although one of these elements is absent in 10–20% of cases [25–27]. PE is a normal placentation disorder characterized by placental ischemia, widespread endothelial dysfunction, and systemic vasospasm. Most cases are diagnosed > 20 weeks of gestation (*early* PE), and in 20–30% of cases post-partum (*late* PE) [26]. The termination of pregnancy is the only confirmed treatment [9]. In a population-based retrospective study in more than one million singleton deliveries, nulliparity, and maternal age ≥ 35 were the major risk factors for PE [28]. Other known factors are obesity, chronic arterial hypertension, pregestational diabetes type 2, and prior PE [29, 30]. The decision and type of delivery should follow the usual obstetric rules; however, expedited delivery is recommended [9].

In non-complicated PE, any sudden deterioration must be promptly detected. Bedside abdominal US plays an important role, because it can identify new liver alterations, presence of peritoneal fluid, or portal vein thrombosis. When the liver is involved, portal thrombosis may be the first imaging sign and suggests severe disease requiring immediate delivery [8, 25]. Other signs of liver involvement are seen if PE progresses to eclampsia and HELLP syndrome. Imaging plays an important role when severe complications are suspected, but delivery should never be delayed when a clinical diagnosis is clearly established.

### Eclampsia and HELLP syndrome

The worsening of PE involves multiple organ damage and is usually associated with eclampsia (defined as new onset of grand mal seizure activity and/or unexplained coma) and HELLP syndrome. The latter is characterized by the triad of hemolysis (H), elevated liver enzymes (EL), and low platelet (LP) count. HELLP occurs in 10–25% of the cases of PE, or 0.8–1% of all pregnancies [12, 25–27]. It develops during the third trimester or early post-partum (up to 4 weeks; 25% of cases) [9]*.* Symptoms range from epigastric, right upper quadrant, or scapular pain to sudden hypovolemic shock [15]. The mortality rate is >5% [8], and a rapid termination of pregnancy is required.

**Fig. 2:**
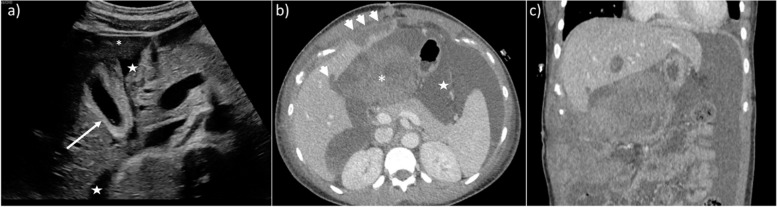
Hepatic rupture in a 27-year-old woman around childbirth. The patient complained of severe right upper quadrant and scapular pain. Alanine aminotransferase and aspartate aminotransferase were augmented, prothrombin time was 30%. Transverse abdominal US scan (**a**) shows the presence of hypoechoic areas (*), free fluid around the liver (stars), and striated thickening of the gallbladder walls (arrow), raising suspicion for pre-eclampsia. Axial (**b**) and coronal (**c**) CT scan on portal venous phase (**b**) demonstrate hepatic lacerations (arrowhead) with surrounding hemoperitoneum and a large peritoneal hematoma (*). Patient underwent emergency cesarean section and liver transplant

It is important to note that Doppler US results show decreased total hepatic blood flow before [31] and at the onset of [32] symptomatic HELLP syndrome. This is probably due to sinusoidal fibrin depositions and obstruction, and disease progression induces liver ischemia, infarction (frequently with fever), periportal necrosis, microthrombi, and/or intrahepatic hemorrhage/hematoma, then capsular lesions including bleeding, and ultimately liver rupture [33, 34]. Although US is the first-line imaging test, CT with or without intravenous contrast administration is the gold standard to detect and follow-up complications [12, 35, 36] (Fig. 2). Studies have reported a marked increase in liver stiffness on US [37], especially during eclampsia [38]; thus, this measurement could be recommended in the future.

**Fig. 3:**
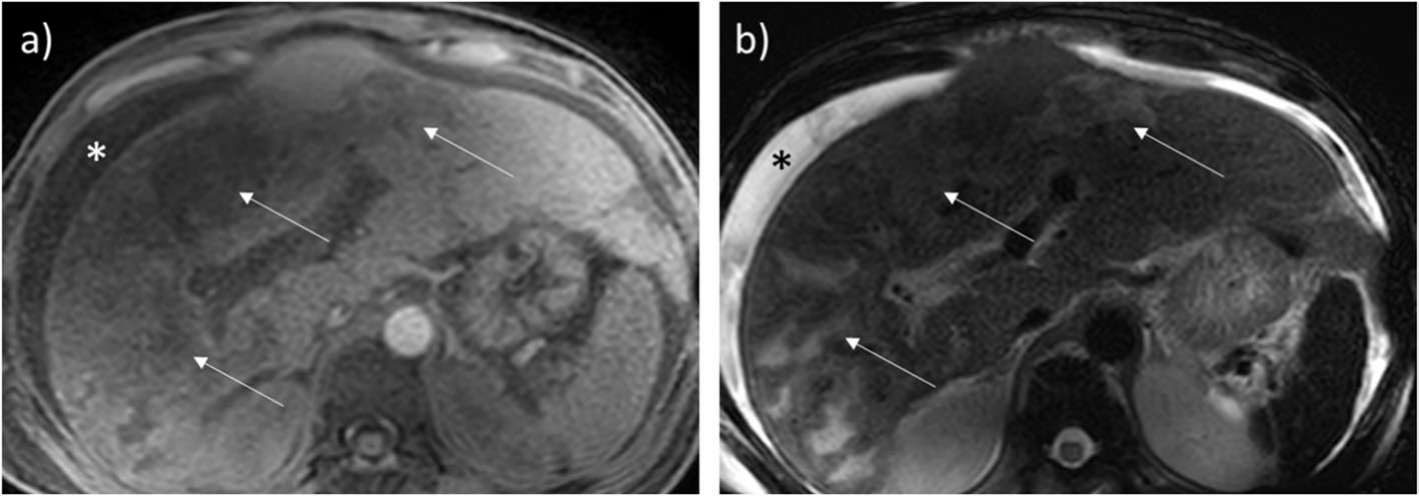
MRI evidence of necrosis due to HELLP in a 32-year-old patient. Necrosis appears as multiple slightly hypointense bands on T1w sequences (**a**), corresponding to areas of T2 hyperintensity (**b**). Ascites is also seen (*)

**Fig. 4:**
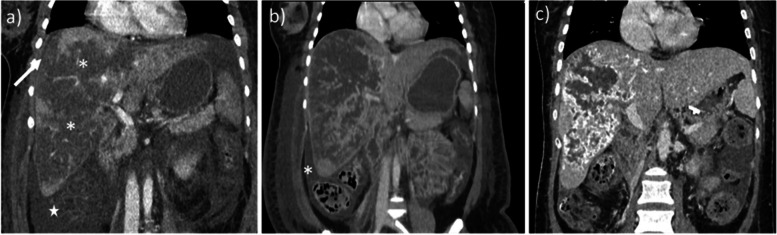
HELLP Syndrome natural history in a 31-year-old woman. Three subsequent scans, all in coronal plane reconstruction, portal venous phase. The first one (**a**) was held at the end of third trimester, when symptoms first appeared. Liver infarctions (*), ascites (star), and a little subcapsular hematoma (arrow) are seen, typical signs of HELLP syndrome. Immediate delivery was promptly performed after the radiologist’s diagnosis. Control CT scans held after 10 (**b**) and 40 days (**c**) show progressive disappearance of subcapsular hematoma and ascites, with calcifications all along the infarcted areas

The imaging findings of HELLP include liver hypertrophy, hyperechoic thickening of the periportal space *(portal halo sign*), thickening of the Glisson capsule and gallbladder wall, ascites, and pleural effusion. On US, probe compression may increase abdominal pain [3, 5, 7, 8]. Infarction is seen as peripheral hypoechoic bands on US, hypo-attenuating, non-enhancing on CT, and ill-defined with a slightly high T2 signal on MRI [15, 33–36] (Fig. 3). Liver hematomas, usually located in the right liver [15], present as heterogeneous, subcapsular lesions, often with hemoperitoneum [15]*.* Intrahepatic hematoma and hepatic rupture are the main life-threatening complications. The latter is seen as a focal irregularity with an adjacent sentinel clot. Foci of active bleeding mirror the attenuation/intensity of the aortic lumen [33] (Fig. 4).

Hemodynamic stabilization is the first step in the management of hepatic rupture and hemoperitoneum, including fluids and blood transfusions, with an emergency delivery. In selected cases, a conservative approach including careful surveillance may be chosen in hemodynamically stable patients [39]*.* In the remaining patients, an urgent laparotomy with liver packing and blood evacuation, hepatic artery ligation, or hepatic arterial embolization is recommended. Partial hepatectomy should be avoided because it worsens the maternal prognosis [40]. When bleeding is uncontrolled or in the presence of rapid deterioration of liver function [27], emergency transfer to a liver transplantation (LT) center is recommended although LT is rarely performed [41].

### Acute fatty liver of pregnancy

**Fig. 5:**
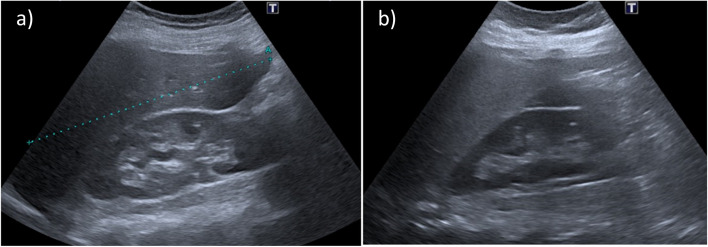
Acute fatty liver of pregnancy (AFLP) in a 32-year-old primiparous woman. US performed in the first month of the pregnancy showed no signs of hepatic steatosis (**a**), while US scan performed at the beginning of third trimester (**b**) demonstrates a markedly increased hepatic echogenicity that, combined with clinical examination, is consistent with acute fatty liver

Acute fatty liver of pregnancy (AFLP) is due to centrilobular microvesicular steatosis, which mainly occurs in the 2nd or 3rd trimester at an incidence of 5/100,000 [42]. AFLP is an obstetric emergency because immediate delivery is required for maternal-fetal survival. Any delay, including non-essential imaging, significantly increases the risk of in-utero or maternal death and is associated with rapid deterioration of liver and kidney functions, disseminated intravascular coagulation and more rarely hemoperitoneum*,* encephalopathy, multiorgan failure, uterine atony, and death [8, 42–45]. The origin of AFLP may be enzyme deficiencies of intramitochondrial fatty acid oxidation in the fetal liver [42]. Symptoms include nausea, vomiting, abdominal pain [42–45], polyuro-polydypsia [45], and rarely, hypertension [9]. Imaging alone is non-specific and may underestimate the diagnosis, especially at the onset of the disease. Thus, the Swansea criteria have been proposed combining 5 clinical symptoms, 5 liver and renal dysfunction laboratory findings, hyperuricemia, bright liver or ascites on US, and microvesicular steatosis on biopsy. Scores ≥ 6/14 confirm the diagnosis [45]. However, the sensitivity for early disease and the specificity in advanced cases are not satisfactory [9]. Imaging findings include a sudden increase in parenchymal US echogenicity (Fig. 5), and liver attenuation ≤ 40 HU on CT [33, 42, 43]. MRI is the most sensitive technique. It provides a qualitative assessment with dual echo T1-weighted sequences [44]—the signal dropout on opposed-phased T1-weighted images can be significant in patients with AFLP [33]—and fat quantification with proton density fat fraction (PDFF) provides precise information on the percentage of fat deposition [46, 47].

## Liver diseases exacerbated by or more frequent during pregnancy

### Acute Herpes Simplex Virus Hepatitis

Both subtypes of herpes simplex virus (HSV) are uncommon causes of symptomatic anicteric acute liver injury. Due to the immunosuppression during the second half of pregnancy, acute hepatitis (AH) due to herpes simplex virus (AHSV) is significantly more frequent in pregnant than in non-pregnant women of the same age [48]. Persistent fever is the main symptom, alone or associated with lesions on the skin or genitals. Serum aminotransferase values vary between 5 to more than 50 times the upper limit of normal. Leukopenia and thrombocytopenia are also highly suggestive of AHSV but are not found in half of the cases at the onset. These features can mimic pre-eclampsia or AFLP and delay these diagnoses [20]. Empirical antiviral treatment with acyclovir should be prescribed as early as possible to avoid progression to acute liver failure (ALF) [49]. Blood RT-PCR of the virus and HSV serologic tests confirm the diagnosis [8, 49, 50]. Mortality reaches 20% [48]. Although imaging can help make the diagnosis it is non-specific (refer to the paragraph “acute hepatitis”).

### Budd-Chiari syndrome

**Fig. 6:**
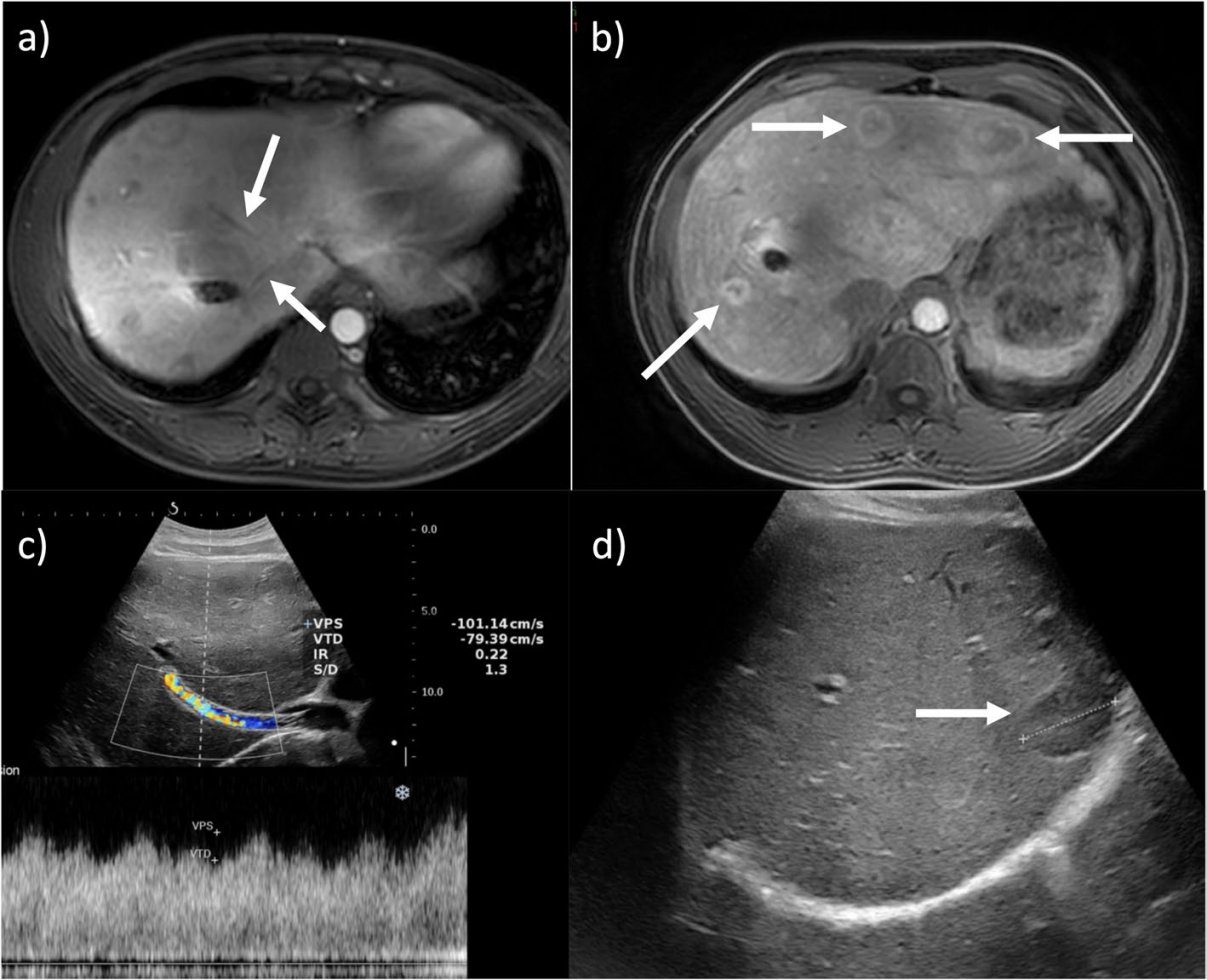
Ultrasound (US) surveillance during pregnancy in a 29-year-old woman with chronic Budd-Chiari syndrome. Pre-pregnancy gadobenate dimeglumine contrast-enhanced MR images (**a**, **b**) obtained during portal venous phase (**a**) and hepatobiliary phase (**b**) show the occlusion of the hepatic veins (**a**—arrows) and multiple benign regenerative nodules with peripheral hyperintensity on hepatobiliary phase (**b**—arrows). Note the presence of a trans-jugular intrahepatic portosystemic shunt (TIPS) connecting the superior vena cava and the portal system. US examination performed during pregnancy (**c**, **d**) allowed to evaluate the patency of the TIPS (**c**), to monitoring the lesion size (**d**) and to eliminate the presence of ascites

Budd-Chiari syndrome (BCS) is a hepatic venous outflow obstruction disorder [33, 51, 52]. Pooled prevalence of pregnancy-related BCS is 13.1% in females with BCS [51]. This suggests that BSC can be triggered by, manifest for the first time, or be aggravated by pregnancy [52, 53] due to the hormonal changes and thrombophilic status during this period [51]. BCS should be suspected in any pregnant woman with persistent abdominal pain, isolated, or associated with the development of ascites and/or hepatomegaly [33, 51, 52, 54]. The risk of decompensation in pregnancy is high and usually manifests by the development of ascites [54]. Doppler US is the first-line diagnostic and screening technique (Fig. 6). When US is inconclusive, contrast-enhanced MRI should be performed [33, 55, 56].

Imaging features include hepatic venous occlusion and an enlarged, heterogenously enhancing liver. In acute cases, a recent thrombus in the inferior vena cava and/or the hepatic veins [12], or a “flip-flopping” enhancement pattern can be seen (early subtle increased enhancement of the periphery, and subsequent prominent central enhancement). Patients with chronic or acute-on-chronic BCS may have a nodular, dysmorphic liver, hepatic venous collaterals, and signs of portal hypertension. Benign (regenerative or FNH-like) nodules are also common. These range from 1 to 4 cm, are homogenously hyperenhancing on hepatic arterial phase, with variable attenuation/intensity on portal venous phase [55, 56], and can increase in size during pregnancy [52]. Anticoagulation is indicated as the first-line therapy for acute disease to improve blood flow, while interventions such hepatic venous angioplasty and TIPS are a second-line treatment. Management should be discussed in expert multidisciplinary centers [54, 57].

### Hereditary hemorrhagic telangiectasia

**Fig. 7:**
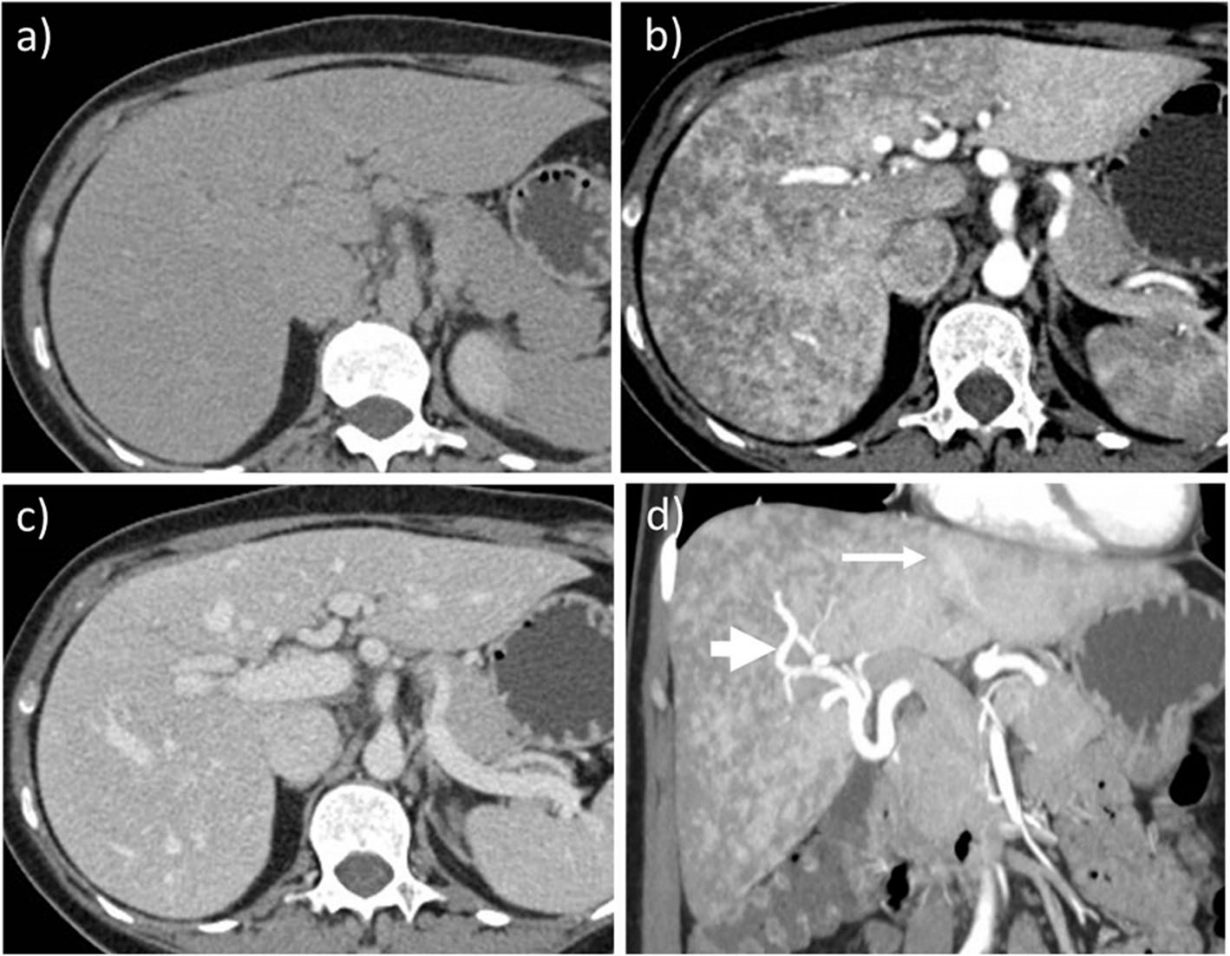
Hereditary hemorrhagic telangiectasia discovered in a 33-year-old pregnant woman. The patient had an episode of hemoptysis that led to a contrast-enhanced CT. Liver is slightly heterogeneous on unenhanced image (**a**). Enhanced CT shows the presence of a heterogeneously enhancing liver on hepatic arterial phase (**b**) with portal phase homogenization (**c**). Coronal plane MIP reconstruction on arterial phase (**d**) shows the presence of enlarged intra- and extrahepatic artery (> 6 mm—thick arrow) and early enhancement of left hepatic vein (thin arrow)

Hereditary hemorrhagic telangiectasia (HHT), or Osler-Weber-Rendu disease, is a rare autosomal dominant vascular dysplasia, characterized by multiorgan telangiectasias and arteriovenous malformations, mainly in the lung and liver [12, 58, 59]. HHT is often diagnosed during pregnancy, mainly in the 3rd trimester or after delivery. Most pregnancies are uneventful, but there is a significant maternal risk of arteriovenous malformation rupture [60]. The Curaçao Diagnostic Criteria are usually used for diagnosis, including the family and personal clinical history associated with findings of diffuse telangiectasias and shunts, heterogeneous liver enhancement, and a common hepatic artery > 6 mm in diameter to confirm the diagnosis [33, 59, 60] (Fig. 7). MRI or contrast-enhanced CT (when bleeding is suspected) are the first-line techniques for suspected HHT in pregnancy. When HHT is known, imaging should be performed to follow the arteriovenous malformations during pregnancy [12, 61]. US or MRI may be the first-line screening technique depending on the location of the aneurysms because the growth of hepatic shunts due to the cardiovascular changes increase the risk of biliary ischemia, bleeding, hemoperitoneum, liver failure, and biliary necrosis [58, 59].

### Hepatocellular adenoma

**Fig. 8:**
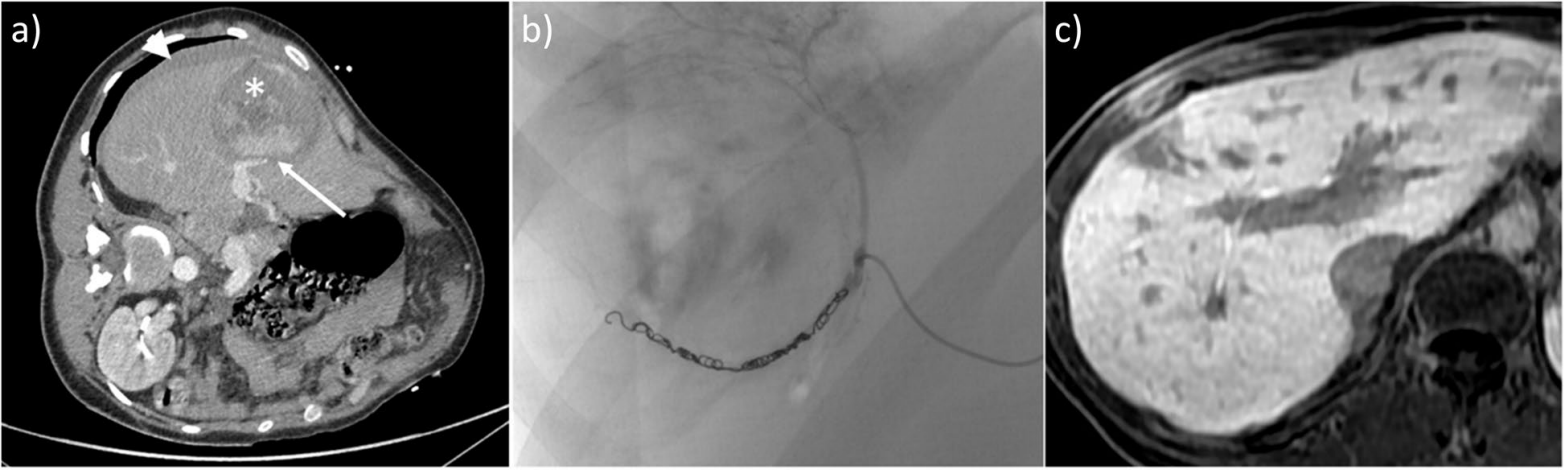
Hepatocellular adenoma evolution during pregnancy in a 29-year-old woman, 39th week of pregnancy, presenting with sudden and strong acute pain in right upper abdomen, associated drop of hemoglobin levels (Hb = 10 g/dL). Axial CT scan on delayed phase (**a**) shows a tumor (*) measuring more than 5 cm, with active bleeding (arrow) and hemoperitoneum (arrowhead). Due to patient instability, arterial embolization combining gelatin sponge injection and selective distal coiling was performed (**b**) followed by fetal extraction. Six years after the event, axial scan, T1w MRI (**c**) shows shrinkage of the lesion

Because of the trophic effect of estrogens, hepatocellular adenoma (HCA) increases in size in 25% of pregnancies [62]. The risk in HCAs < 5 cm are minimal while in HCAs > 5 cm the risk of intratumoral bleeding, spontaneous rupture, and hemoperitoneum is high (Fig. 8) [62]. Monitoring is recommended with ultrasound examinations each trimester [63]. Teamwork is essential in the evidence of growth or bleeding, the latter requiring trans-arterial embolization or surgery in some patients. It is hypothesized that during pregnancy, the subtypes more prone to bleeding are the inflammatory or Sonic Hedgehog subtypes, as seen in the general population [64]. Imaging will mirror that of the general population. The Sonic Hedgehog subtype does not have any specific imaging characteristics but is more frequent in obese patients. The inflammatory subtype can present with strong arterial enhancement, persisting in the portal and late venous phase for both CT and MRI. On MRI, the presence of the “atoll sign,” with a ring of T2 hyperintensity surrounding an isointense lesion, increases the specificity [64]. The lesion-to-liver contrast enhancement ratio on hepatobiliary phase may help differentiate these subtypes from the beta-catenin activated subtype [65].

### Portal vein thrombosis

**Fig. 9:**
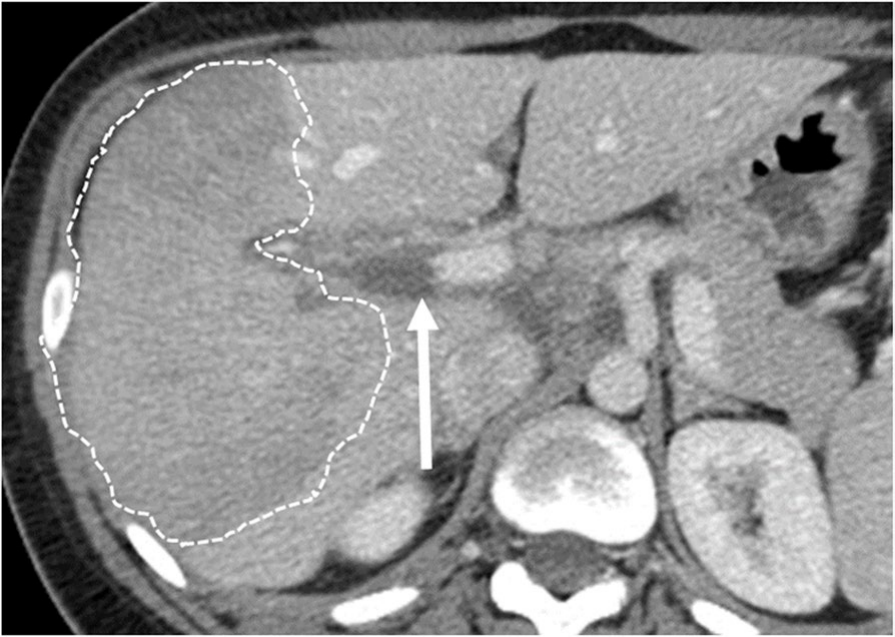
Pre-eclampsia (PE) outcomes in a 24-year-old primiparous woman. This patient did not perform imaging studies during pregnancy, but presented with persistent right upper quadrant pain around delivery, increased AST/ALT and arterial blood hypertension; hence, PE was suspected. Axial, portal venous phase CT scan performed after childbirth demonstrates portal vein thrombosis (arrow) and parenchymal infarction (dotted lines)

Portal vein thrombosis (PVT) not associated with well-known hepatic risk factors is rare during pregnancy or puerperium [66]. In a minority of cases PVT is acute with sudden abdominal pain and often, fever [66]. Acute PVT is characterized on US by hyperechoic appearance and on CT by a spontaneous hyperattenuating appearance with post-contrast filling defects. There is a risk of bowel ischemia in the presence of extension into the mesenteric veins. Anticoagulant therapy (with low molecular weight heparin) is required. Short-term Doppler US follow-up must be performed to evaluate disease progression or response to treatment. Chronic PVT is identified as a complication of portal hypertension, HELLP syndrome, PE, eclampsia, or by chance [66]. Prothrombotic factors must be searched for in all cases of PVT. The outcome of pregnancy is good after 20 weeks of gestation, with 58% of births after 36 weeks. Complications include miscarriage (20%) and preterm births [53, 66] (Fig. 9).

### Cholelithiasis

Hyperestrogenemia, hypercholesterolemia, and decreased gallbladder mobility result in supersaturated bile during pregnancy [8, 33] and 10% of pregnant women develop gallstones or sludge [7]. Imaging findings and complications are similar to those in the non-pregnant population, including cholecystitis, choledocholithiasis, and pancreatitis. While US is the first-line imaging technique for suspected gallstone disease, MRCP is safe [20, 33]. Supportive care is the initial management strategy, but endoscopic retrograde cholangiopancreatography (ERCP) has a well establish therapeutic role, and may be performed especially in the second trimester [20]. Conservative management can result in more frequent recurrent biliary symptoms, acute episodes of pancreatitis, hospitalizations, and cesarean sections. Each case should be discussed with the obstetrician, especially in the first trimester. Studies on ERCP have shown that radiation exposure is usually < 6 mGy and should therefore be considered safe [67].

## Coincidental liver diseases

### Acute hepatitis

AH should be considered in the presence of sudden jaundice and acute liver injury or failure in pregnancy [50, 68–70]. Although viral hepatitis (virus A to E) is the most common cause of jaundice in pregnancy worldwide [50], hepatotoxicity due to drug reactions, herbal or dietary supplements should be ruled out [63–69]. In India, the hepatitis E virus is a common cause of AH in pregnancy with a 25% mortality rate [8, 50], while acetaminophen overdose is responsible for 30% of the cases of ALF in pregnancy in the USA [68].

Autoimmune hepatitis may also be seen in pregnancy, requiring rapid diagnosis and therapy. Imaging can help in the diagnosis of AH with a combination of hepatomegaly, gallbladder wall thickening, and periportal edema [33]. AHSV should be suspected in the presence of multiple small (< 1 cm) nodular lesions, between 1 and 3 mm, that are hypoechoic on US, hypo-attenuating on CT, and represent necrotic foci [70].

### Incidental focal liver lesions

**Fig. 10:**
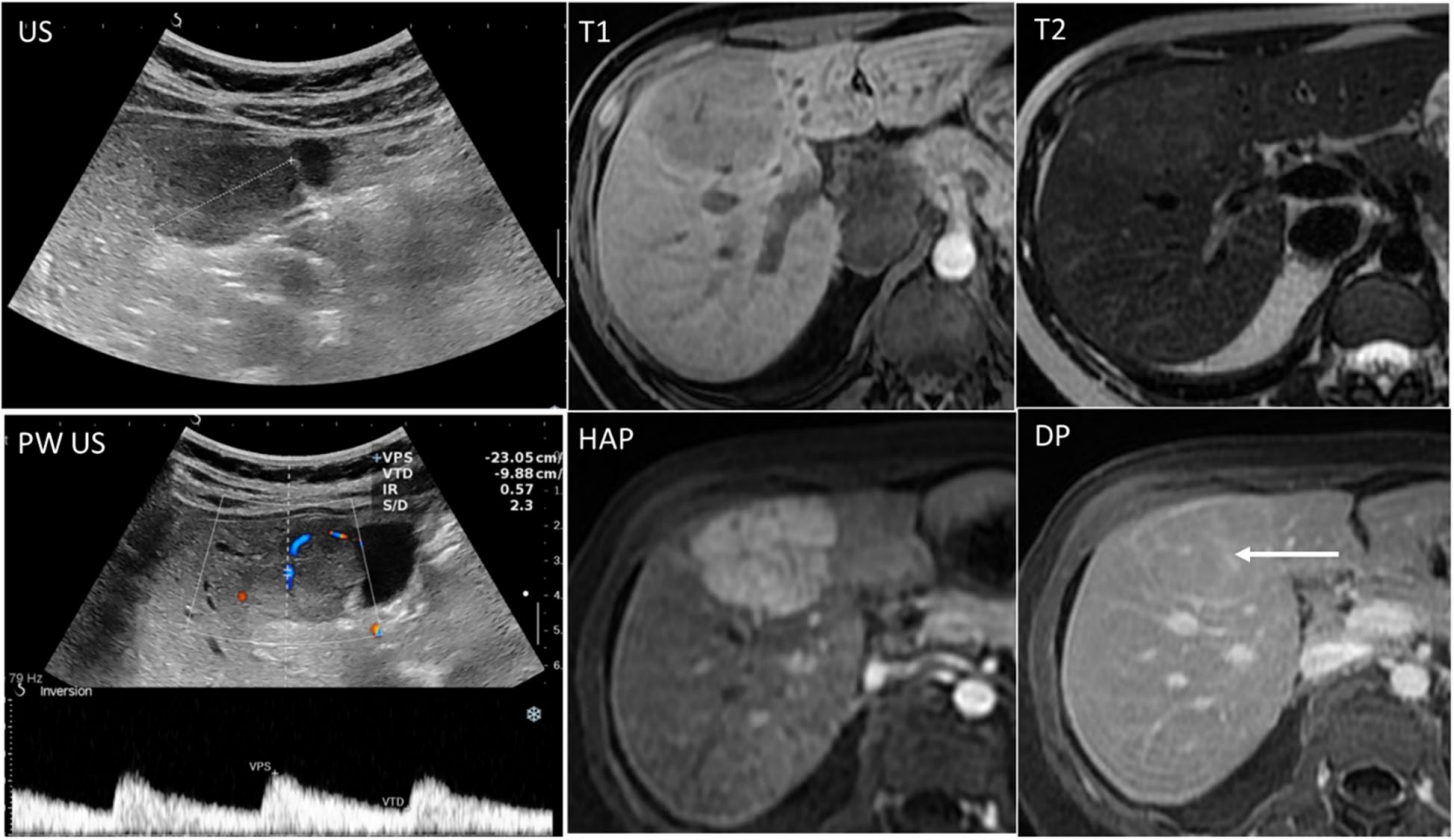
Focal liver lesion management in a 27-year-old pregnant woman. During routine US, a liver mass is seen (first picture, upper row), showing vascularization and arterial flow on Doppler US (first picture, lower row). MR study was requested and demonstrates a T1 hypointense lesion, slightly hyperintense on T2. This lesion was not deemed malignant therefore contrast-enhanced MRI was withheld until childbirth and showed avid enhancement on hepatic arterial phase (HAP) with homogenization on delayed phase (DP) and a central scar (arrow), typical of focal nodular hyperplasia

Like in the general population, an incidental liver lesion without no underlying liver disease is benign in 96% of the cases [71]. If the pre-test probability and unenhanced imaging support a benign diagnosis, further characterization with contrast administration should be performed after childbirth (Fig. 10). However, CEUS is safe for single lesions during pregnancy [11].

**Fig. 11:**
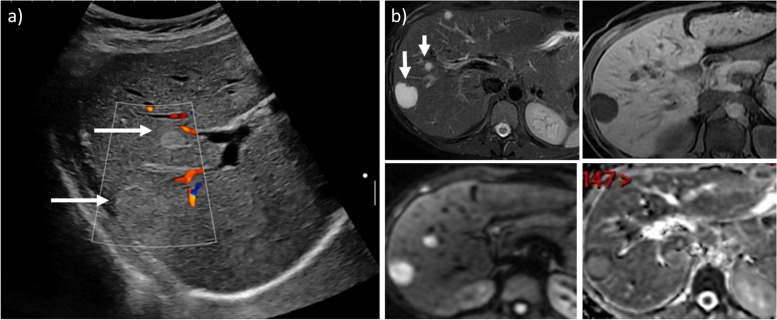
During a routine US (**a**), hyperechoic hepatic lesions (arrows) were seen in a 37-year-old pregnant woman. These lesions showed no vascularization on Doppler US nor stiffness, and the liver had no signs of chronic liver disease, so they were classified as hemangiomas. Months later, one lesion slightly grew, and an unenhanced MR was performed (**b**), showing high T2 signal with low T1 signal (upper row) and no diffusion restriction (lower row), thus confirming the diagnosis of hemangiomas

During or after pregnancy, hemangiomas can grow (Fig. 11) [72, 73] but they are indolent. Spontaneous hepatic rupture is exceptional [73]. Focal nodular hyperplasia (FNH) is asymptomatic, does not grow, and does not require follow-up [73, 74].

If an unknown lesion presents fat, HCA is suggested. HCAs have non-specific imaging characteristics on US and CT; therefore, MRI should be performed. If malignancy is suspected, breast cancer should be suspected as it is the most common primary cancer in pregnancy [75], and is characterized by hypoenhancing liver metastases. Cystic components may suggest a mucinous gastrointestinal or ovarian primary, while hyperenhancing lesions suggest a metastatic choriocarcinoma [76], sarcoma, and renal or neuroendocrine tumor. US surveillance is recommended each trimester in pregnant patients with a history of extrahepatic cancers known to metastasize to the liver [63]. Biopsy may also be considered whenever needed in incidental focal lesions, severe liver dysfunction in transplanted patients, or in cases of severe liver disease. Liver biopsy appears to be safe and well tolerated in the first trimester. It is difficult to draw firm conclusions in other cases, which should be judged based on the risk and benefit of this procedure [77].

### Metabolic dysfunction–associated steatotic liver disease

Metabolic dysfunction–associated steatotic liver disease (MASLD) (previously known as non-alcoholic steatohepatitis; NAFLD) is currently the most frequent chronic liver disease worldwide [78] and includes a spectrum from simple steatosis to hepatocellular injury and fibrosis. The prevalence of MASLD in pregnancy has nearly tripled in the last decade and is independently associated with a higher incidence of hypertensive complications, post-partum hemorrhage, pre-eclampsia HELLP syndrome, and preterm birth [79–81]. Treatment in pregnancy mainly involves nutritional measures and physical activity [78].

Imaging can determine the presence of liver steatosis. PDFF-MRI is the most accurate biomarker, and the fibrosis can be estimated on transient elastography, US-, or MR-elastography [82, 83]. A differential diagnosis is made with AFLP using clinical-laboratory data and the absence of other findings connected with AFLP. Although the mechanisms by which MASLD favor adverse maternal and perinatal events are not well defined, the presence of MASLD warrants preconception counselling and management by specialized obstetricians [81].

### Cirrhosis

**Fig. 12:**
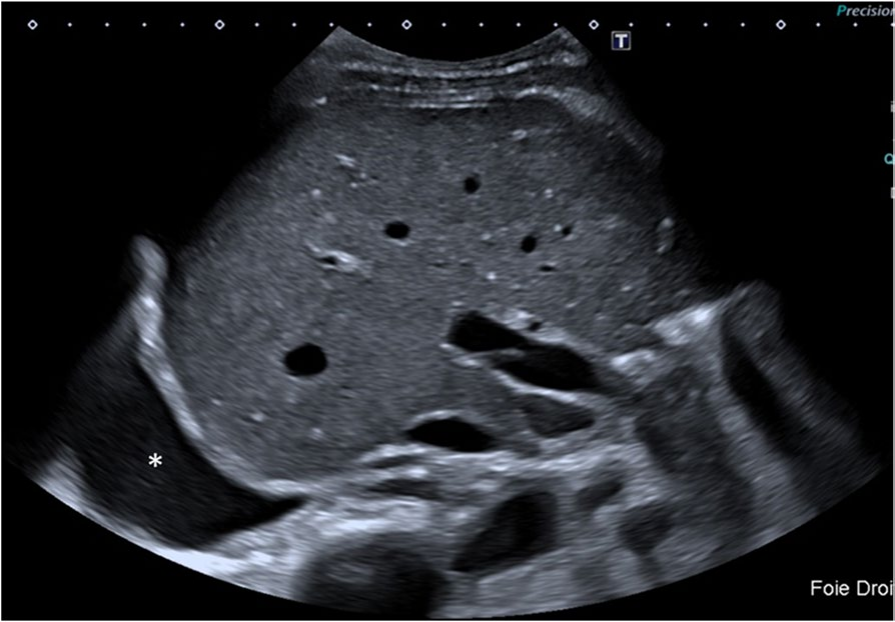
Cirrhotic decompensation during an unplanned pregnancy in a 31-year-old patient with hepatitis B virus-related cirrhosis. Surveillance abdominal US scan during the second trimester shows the presence of pleural effusion (*) and mild ascites (*). The therapy of the patient was therefore changed accordingly, and the gestation was otherwise uneventful

Chronic viral hepatitis (B and C), metabolic dysfunction–associated steatohepatitis (previously known as non-alcoholic steatohepatitis), autoimmune diseases, and alcohol are the main causes of cirrhosis in pregnancy [79, 80]. Pre-conceptional cirrhosis compensation is the mainstay of successful pregnancies [84]**.** During pregnancy, hepatic decompensation, ascites, and variceal hemorrhage can occur. The risk of decompensation can be predicted by scores such as the MELD (model for end-stage liver disease), the preconception Albumin-Bilirubin score (ALBI), or preconception aspartate aminotransferase to platelet ratio index (APRI) [85]. Variceal bleeding remains the most frequent result of cirrhotic decompensation and death in pregnancy [86] and preventive endoscopic band ligation is recommended [87, 88]. Close monitoring is required in all trimesters (Fig. 12) to assess ascites, portal vein thrombosis, and new or growing focal lesions. Spontaneous rupture of a splenic artery aneurysm is a rare event found in 0.00001–0.003% of cases [89]. Maternal and fetal survival is 26% in the mother and 50% in the fetus and depends on a rapid diagnosis and effective treatment. It mainly occurs in the second half of pregnancy or during labor [89, 90]. Pregnancy, hyperdynamic circulation, and hormone-induced alterations of the arterial walls are contributing factors. Abdominal imaging with CT or MRI helps the diagnosis, showing the size and location of the aneurysm and its rupture. Urgent embolization of the splenic artery or laparotomy (with delivery) must be performed for uncontrolled bleeding [89–91].

### Hepatocellular carcinoma

Hepatocellular carcinoma (HCC) is very rare in pregnancy, but the prognosis is worse than in the non-pregnant population, with a 1-year survival rate < 30%. Rapid and aggressive tumor growth is described, with a higher chance of rupture, probably due to estrogens and neovascularization [92–94]. Surveillance with US or MRI is recommended during each trimester in women at risk of HCC, [63]. When HCC is suspected, characterization by either contrast-enhanced MRI or CT should not be delayed [92]. Surgery is usually recommended, but loco-regional treatments such as percutaneous tumor ablation have been found to be safe [92–95].

Fibrolamellar hepatocellular carcinoma is an uncommon variant of HCC that mainly occurs in young adults and is rarely observed in pregnancy. Imaging is crucial for a differential diagnosis with focal nodular hyperplasia. While both these events share hyperenhancement and a central scar, only HCC shows portal or delayed phase wash-out or presents with a mosaic architecture. Other typical findings suggesting fibrolamellar HCC are the presence of a central calcification, in which is rare in an FNH, and lesion heterogeneity [96].

### Liver abscesses and parasitosis

Pregnant patients with sepsis and fever who undergo abdominal US should always be checked for liver abscess [97]. The most common causes in pregnancy include *E. Coli*, *Bacterioides spp*., and *Entamoeba histolytica* [97, 98]. Maternal and fetal mortality is high for liver abscesses in these cases since pregnancy and immunodepression are risk factors for invasive infections, septic shock, and/or liver rupture [97–99]. Imaging is important for both diagnosis and surveillance. US shows a thick-walled hypoechoic lesion, with peripheral and progressive ring-like enhancement on CEUS. Unenhanced MRI shows a T2-hyperintense solid-fluid lesion with central diffusion restriction [98]. Further characterization can be obtained by US-guided aspiration [100]. The symptoms and complications of hepatic echinococcosis may be severe during pregnancy and may therefore require urgent treatment [101].

## Liver transplantation considerations

In women with prior liver transplantation (LT) pregnancies that are carefully planned and monitored are successful in 80% of cases [102]. Drugs linked to fetal toxicity should be stopped at least 6 months before conception in any partner [7]. During pregnancy, radiologists should consider that LT women are at an increased risk of infections, pre-eclampsia, eclampsia, HELLP syndrome, and preterm delivery [102]. Acute graft rejection and liver failure can occur even during pregnancy and must be carefully and promptly detected. Liver Doppler US is the first imaging technique to monitor the transplant and pregnancy-related complications. When inconclusive, non-contrast MRI is preferred to CT. The literature also suggests that LT may be safely performed during pregnancy [103, 104].

## Diagnostic algorithms

### Asymptomatic patients with unknown focal liver lesions

If an unknown focal liver lesion is incidentally seen on US, the clinical setting and characteristics of the lesion should be considered first. In particular:

1. Cystic lesions: anechoic lesions, no walls, inner components, or septa suggesting simple hepatic cysts. If multiple and small, consider biliary hamartomas. If the walls are thick or with calcifications, abscesses, echinococcosis, or, rarely, cystic metastases are differential diagnoses. When in doubt, perform MRI.
2. Solid lesions:
  - Hyperechoic: the most common entities are hemangiomas and focal steatosis, followed by FNHs, HCAs, and metastases. With no previous images or if uncertain, consider MRI or CEUS. If unenhanced MRI suggests a benign lesion, definitive characterization could be postponed. Focal steatosis follows a typical distribution pattern around the gallbladder and close to the hilum.
  - Hypoechoic: US is not specific; consider CEUS (if single), or MRI. Contrast administration only if suspected malignancy. Regular US follow-up is needed for HCAs (> 5 cm risk of bleeding). Multiple, small hypoechoic spots suggest metastases, infections, or sarcoidosis. With periportal edema and gallbladder wall thickening, consider hepatitis.
  - Isoechoic: FNH. Color Doppler might show the central artery and CEUS the typical spoke-wheel pattern. FNH can only be suspected on unenhanced MRI.

If necrotic/liquid components are present, consider metastases and abscess.

**Fig. 13:**
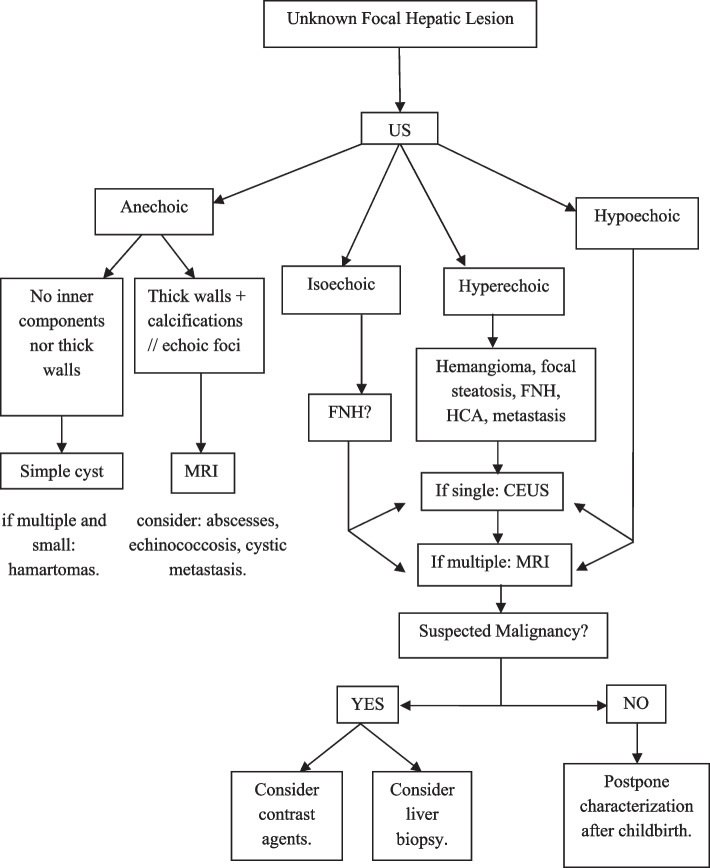
Diagnostic algorithm presenting the management and differential diagnoses to consider in an asymptomatic pregnant woman with focal liver lesions. abbreviations: US = Ultrasonography; MRI = Magnetic Resonance Imaging; CEUS = Contrast Enhanced US; FNH = Focal Nodular Hyperplasia; HCA = Hepatocellular Adenoma

In the presence of fat, consider HCA or atypical FNH. In the presence of cirrhosis, siderotic content is a sign of regenerative or dysplastic nodules. Hematic nodules include hematomas or bleeding HCAs. Contrast-enhanced MRI or CT should be performed when active bleeding is suspected. Figure 13 contains a diagnostic chart for characterization of focal liver lesions in pregnancy.

### Urgent or emergency management

In stable patients with right upper quadrant pain, bedside US is usually the first-line examination. If abdominal pain, fever, and ALT or AST >10 times the upper normal value are present, HSV hepatitis should be suspected and acyclovir therapy should start empirically as soon as possible. If leukopenia is seen too, the diagnosis is almost certain. Differential diagnosis include hepatic bleeding and hematoma. Hyperleukocytosis will raise concern for liver abscess.

If hepatic infarction, rupture, hemorrhage, or hematoma are suspected, CT or MRI should be performed with contrast agents, if necessary, without delay. HELLP syndrome, liver tumors, trauma, or AFLP may be complicated by hepatic infarction, bleeding, hematoma, or liver capsule rupture, with a risk of maternal death > 5%. After the diagnosis, a decision of immediate or delayed delivery depends on both the cause of hemoperitoneum and the term of pregnancy and should be made by an expert multidisciplinary team.

**Fig. 14:**
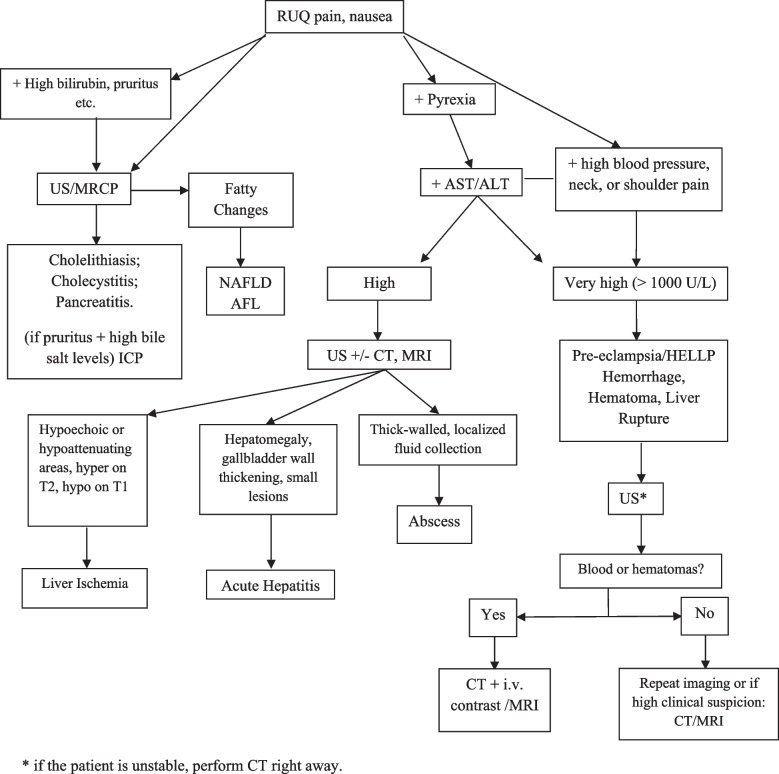
Diagnostic Algorithm for liver urgencies and emergencies in pregnancy. abbreviations: RUQ = Right Upper Quadrant; ICP = Intrahepatic Cholestasis of Pregnancy; AFLP = Acute Fatty Liver of Pregnancy; HELLP = Elevated Liver Enzymes, Low Platelet; US = Ultrasonography; MRI = Magnetic Resonance Imaging; MRCP= Magnetic Resonance Cholangiopancreatography; CT = Computed Tomography; MRI = Magnetic Resonance Imaging; AST = Aspartate transaminase; ALT = alanine aminotransferase (ALT); NAFLD = Non-Alcoholic Fatty Liver Disease

In the presence of persistent signs of hemodynamic instability, immediate blood transfusions and delivery are required prior to trans-arterial embolization or surgery. Coordination with an LT center is recommended. Figure 14 provides a diagnostic chart for urgent and emergency situations.

## Conclusion

The global incidence of LD in pregnancy is rising. Acute LD in pregnancy may require urgent care. It is highly important to detect clinical syndromes and hepatic lesions that require urgent care or delivery as rapidly as possible. In pregnant women, abdominal imaging is crucial especially for the diagnosis of emergencies, like bleeding, hematomas, and liver rupture in pre-eclampsia/HELLP, or the rupture of splenic artery aneurysm in cirrhotic women. Liver diseases will be best managed when there is a close cooperation between the obstetrician, the radiologist and the hepatologist, to avoid adverse consequences on both mother and child.

## Data Availability

Data sharing is not applicable to this article as no datasets were generated or analyzed during the current study.

## Abbreviations

AFLP: Acute fatty liver of pregnancy
AH: Acute hepatitis
AHSVH: Acute herpes simplex virus hepatitis
ALF: Acute liver failure
BCS: Budd-Chiari syndrome
CDUS: Color Doppler ultrasonography
CEUS: Contrast-enhanced ultrasounds
CT: Computed tomography
FNH: Focal nodular hyperplasia
HCA: Hepatocellular adenoma
HCC: Hepatocellular carcinoma
HELLP: Hemolysis, elevated liver enzymes, low platelets syndrome
HEV: Hepatitis E virus
HHT: Hereditary hemorrhagic telangiectasia
HSV: Herpes simplex virus
ICP: Intrahepatic cholestasis of pregnancy
MASLD: Metabolic dysfunction–associated steatotic liver disease
MRCP: Magnetic resonance cholangiopancreatography
MRI: Magnetic resonance imaging
NAFLD: Non-alcoholic fatty liver disease
PDFF: Proton density fat fraction
PE: Pre-eclampsia
PVT: Portal vein thrombosis
US: Ultrasounds
